# Case Report: Using basic liver function tests as a guide to suspected Wilson’s disease

**DOI:** 10.12688/f1000research.54569.2

**Published:** 2022-03-03

**Authors:** Sudeep Adhikari, Prashant Kumar Shah, Yuba Raj Sharma

**Affiliations:** 1 Department of Internal Medicine, Pyuthan Hospital, Pyuthan, 22300, Nepal; 2 Department of Internal Medicine, Patan Academy of Health Sciences, Kathmandu, 26500, Nepal

**Keywords:** Wilson's disease, liver disease, basic liver function tests, Nepal

## Abstract

We present a case of a 36-year-old female patient who presented with subacute liver disease with a history of alcohol abuse. On basic liver function tests (LFT), she had aspartate transaminase / alanine transaminase > 2.2 and alkaline phosphatase / total bilirubin < 4. This pattern in acute liver failure patients signifies Wilson’s disease. Its presence in our patient with subacute liver disease also prompted us to suspect Wilson’s disease and we extended the liver disease screen to include slit lamp eye examination for Kayser-Fleischer rings, serum ceruloplasmin and 24-hour urinary copper level, which led to the diagnosis. She improved clinically and biochemically with zinc acetate therapy.  As screening for rare diseases is not always possible in low-income countries, this case demonstrates the usefulness of the basic LFT as a guide for suspecting Wilson’s disease in patients with liver disease.

## Introduction

Wilson’s disease is an autosomal recessive disorder of copper metabolism that leads to liver disease and neuropsychiatric manifestations. The majority of patients are diagnosed between the ages of five and 35 years. This disease is usually suspected in patients with liver disease of uncertain etiology, especially if accompanied by neuropsychiatric symptoms. But it can also present with liver abnormalities alone. Untreated, this disease is universally fatal, with patients dying from cirrhosis or progressive neurologic disease. ^1^ Hence its timely recognition is important. It is not usually diagnosed in resource-limited countries as diagnostic tests are not easily available. Here we present a case of Wilson’s disease from Nepal. The patient presented with liver disease (without neuropsychiatric features), and we discuss how basic liver function test parameters can be used as a guide to suspected Wilson’s disease.

## Case

A 36-year-old woman from Lalitpur, Nepal presented to the emergency department of Patan Hospital in December 2018, with complaints of abdominal distension and yellowish discoloration of the skin for two months and shortness of breath for the last 10 days. She had no history of fever, cough, abdominal pain, nausea, vomiting, black stools, altered sensorium or abnormal body movement. She had a history of consuming home-made alcohol, almost daily for 12 years, although quantification of the intake could not be done.

On examination she appeared icteric and pale. Blood pressure was 90/60 mm Hg, pulse 78 beats per minute, respiratory rate 20 breaths per minute, temperature 98.7 Fahrenheit and oxygen saturation of 97% at room temperature. Abdomen was distended and non-tender, liver and spleen were not palpable and shifting dullness was present. Chest examination showed decreased air entry. Bilateral pitting pedal edema was noted. Neurological examinations did not reveal any abnormality. She was looked after at another health care center three weeks prior to her admission as a case of alcoholic liver disease. She stopped drinking alcohol, but without improvement in her symptoms. Her investigations are as shown in Table 1.

**Table 1.** Laboratory parameters of the patient before and after treatment for Wilson’s disease.

Parameters	Three weeks prior to admission (November 2018)	Two weeks prior to admission (December 2018)	Day of admission (December 2018)	One month later (January 2019)	11 months later (November 2019)	Normal range
White Cell Count (WCC)	36600	-	10400	-	6100	4000-11000/microL
Hemoglobin	8.1	-	7.8	9.5	10.1	11.5-15 g/dl
Platelet	150000	-	150000	-	150000	150000-400000/microL
Reticulocyte	-	-	6	-	-	0.5-1.5%
Glucose (Random)	-	-	107	-	-	70-140 mg/dl
Urea	12	-	26	44	12	15-36 mg/dl
Creatinine	0.7	1.5	1	1.1	0.6	0.7-1.2 mg/dl
Sodium	127	-	128	136	136	135-145 mmol/L
Potassium	2.9	-	3.2	3.9	3.3	3.5-5 mmol/l
Total bilirubin	34.1	30.2	21.3	4.9	1.6	0.2-1.3 mg/dl
Direct bilirubin	21.9	22.0	10.9	0.1	0.5	0-0.4 mg/dl
Indirect bilirubin	12.2	8.2	10.4	4.8	1.1	0.2-0.8 mg/dl
Aspartate transaminase (AST)	144	79	442	78	42	15-43 IU/L
Alanine transaminase (ALT)	40	37	127	54	27	13-72 IU/L
Total protein	4.9	5.1	5.2	-	-	6-8 g/dl
Serum albumin	2.0	1.6	2.1	-	-	3.5-5 g/dl
Alkaline phosphatase (ALP)	83	-	66	83	100	38-126 U/L
International Normalized Ratio (INR)	2.64	-	2.39	-	-	

Ultrasound of the abdomen showed hepatosplenomegaly and gross ascites. Ascitic fluid tap was done and sent for analysis, which showed WCC 100 cells/μL (neutrophils 60% and lymphocytes 40%), protein 1 gm/dl and albumin 0.5 gm/dl. Upper gastrointestinal endoscopy showed small sized esophageal varices and mild portal hypertensive gastropathy. Hepatitis B surface antigen (HBsAg) and hepatitis C antibody were nonreactive. Direct Coombs test was negative. Patient was started on furosemide 20 mg once daily, spironolactone 50 mg once daily and thiamine 100 mg once daily.

Slit lamp examination of her eyes showed the presence of Kayser–Fleischer (KF) rings. Twenty-four-hour urinary copper was 85.70 (normal < 60) μg and serum ceruloplasmin level was 23 (normal 20–60) mg/dl. The diagnosis of subacute liver failure with Wilson’s disease was made. We started oral zinc acetate (50 mg elemental zinc thrice daily) along with the above drugs and she was discharged in one week of admission. There was clinical and biochemical improvement as seen during one-month (January 2019) and 11 months (November 2019) follow-up visits ( Table 1).

## Discussion

This female patient who presented with features of chronic liver disease had a history of alcohol intake for almost 12 years. Nepal is a low-income country in South Asia, and a full chronic liver disease screening (including tests for Wilson’s disease, autoimmune hepatitis, hemochromatosis) is usually not possible in the Nepali health setup. The limited screen includes alcohol history, history of any drug or toxin use and serologies for hepatitis B and C. ^2^ However, the people mostly consume home-made alcohol in Nepal, and the exact quantification is usually not possible to estimate whether the amount of intake is significant enough to cause liver disease or not. ^3^ We usually ascribe liver disease in any patient to the alcohol, no matter the amount of intake by the patient, especially if serologies are nonreactive and there is no drug or toxin exposure. In our patient with a history of alcohol use, we extended the tests beyond the conventional limited screen, which led to a diagnosis of Wilson’s disease.

Basic liver function tests (LFT) including bilirubin, transaminases level and alkaline phosphatase (ALP) are available in most of the health centers in Nepal. In any patient with liver disease who presents with acute liver failure, the combined ratio of ALP/total bilirubin (<4) and aspartate transaminase (AST)/alanine transaminase (ALT) (>2.2) provides sensitivity and specificity of 100% for diagnosing Wilson’s disease. ^4^ In our patient, ALP/total bilirubin was 3.09 and AST/ALT was 3.4 during the admission ( Table 2). Although she did not have acute liver failure during the presentation, we used this pattern of LFT (the ‘Wilson’s pattern’) as a guide to suspect the possibility of Wilson’s disease and ordered slit lamp examination of eyes for KF rings, serum ceruloplasmin level and 24-hour urinary copper tests.

**Table 2.** Wilson’s pattern of liver function tests of the patient before and after treatment. (alkaline phosphatase (ALP), aspartate transaminase (AST), alanine transaminase (ALT)).

	ALP/total bilirubin	AST/ALT
Three weeks prior to admission (November 2018)	2.43	3.6
Day of admission (December 2018)	3.09	3.4
One month later (January 2019)	16.9	1.4
11 months later (November 2019)	62.5	1.5

The diagnosis was made using the Leipzig scoring system for Wilson’s disease. ^5^ Total points obtained were four (two for KF ring, one for Coombs negative hemolytic anemia (anemia with increased reticulocyte percentage and increased indirect fraction of bilirubin), one for high 24-hour urinary copper), which established the diagnosis of Wilson’s disease. However, she had no neurologic symptoms and her serum ceruloplasmin level was normal. Liver copper and mutation analysis were not performed.

The preferred treatment choice for patients with Wilson’s disease with cirrhosis is copper chelator like D-penicillamine or trientene initially, followed by oral zinc therapy in maintenance. However, the treatment with zinc acetate was started in this case because of unavailability of chelating agents. On follow up in one month, she had no abdominal distension and jaundice. LFTs were improved, which continued to improve till 11 months follow up ( Table 1). Even the ‘Wilson’s pattern’ of LFTs was resolved with treatment ( Table 2).

## Conclusion

LFTs are simple and readily available tests even in remote parts of Nepal, done in all patients with liver disease. As screening for rare diseases is not always possible in low-income countries, this case demonstrates the usefulness of the ‘Wilson’s pattern’ LFT as a guide for suspecting Wilson’s disease in patients with liver disease. However, the sensitivity and specificity of this pattern is yet to be studied in patients with subacute liver disease.

## Data availability

All data underlying the results are available as part of the article and no additional source data are required.

## Consent

Written informed consent for publication of the clinical details and/or clinical images was obtained from the patient.
