# Supplementary material for: Case Report: Using basic liver function tests as a guide to suspected Wilson’s disease
Source: F1000Res. 2022 Mar 3;10:608. Originally published 2021 Jul 20. [Version 2] doi: 10.12688/f1000research.54569.2 (PMC9010801; doi:10.12688/f1000research.54569.2)
Supplement: Peer review history

# Peer review: Reviewer response for version 2

**DOI:** 10.5256/f1000research.120012.r126145

**Published:** 2022-04-14

**Authors:** Raffaele Iorio

**Affiliations:** 1 Department of Translational Medical Science, Section of Pediatrics, University of Naples Federico II, Naples, Italy

The revised version is improved.

Are enough details provided of any physical examination and diagnostic tests, treatment given and outcomes?

Partly

Is the case presented with sufficient detail to be useful for other practitioners?

Yes

Is sufficient discussion included of the importance of the findings and their relevance to future understanding of disease processes, diagnosis or treatment?

Partly

Is the background of the case’s history and progression described in sufficient detail?

Partly

Reviewer Expertise:

Liver disease, Wilson' disease, pediatric liver disease

I confirm that I have read this submission and believe that I have an appropriate level of expertise to confirm that it is of an acceptable scientific standard.

# Peer review: Reviewer response for version 1

**DOI:** 10.5256/f1000research.58069.r100231

**Published:** 2021-12-09

**Authors:** Jason L. Burkhead

**Affiliations:** 1 Department of Biological Sciences, University of Alaska Anchorage, Anchorage, AK, USA

Wilson Disease is considered a rare disease, though recent studies indicate that specific populations may have significantly greater prevalence than the generally accepted 1:30,000 (e.g. estimated 1:3,000 for S. Korea). It is also accepted that Wilson Disease can be challenging to diagnose. The authors of this case report that after an initial diagnosis of alcoholic liver disease and symptoms that did not improve after cessation of alcohol consumption, further etiologies were considered. Notably, the authors applied the “Wilson Pattern” of liver function tests as an initial motivation to specifically assess Wilson Disease, including slit lamp examination and parameters that can be used in the Leipzig Score.

 The overall message of this report is that the Wilson pattern of liver function tests is important in diagnosis, particularly in developing countries that may be resource-limited. This message is not particularly novel, but it is valuable to highlight. The Wilson pattern was developed based on patients with acute liver failure, however. It is not yet known whether the specificity and sensitivity of the test is 100% in subacute liver disease—as the authors note under the Conclusion section. This observation as well as the qualifier are useful to highlight and enhance the importance of the report.

 It appears from the report that the choice to use Zn was made due to the lack of chelator availability. The report indicates “50 mg elemental zinc thrice daily.” It is expected that a zinc salt was prescribed, where it would be important to report the specific salt used. The observation that the patient’s liver function tests improved with zinc supplementation is promising and may be useful to others. Given the apparent ability to assess 24-hour urinary Cu, it would be interesting to know if this value changed with treatment.

 In summary, this is an important case report in that it promotes consideration of liver function tests in suspicion of Wilson Disease, which is particularly valuable in locations where specialized diagnostic resources are limited. The apparent successes in the case are also notable.

Are enough details provided of any physical examination and diagnostic tests, treatment given and outcomes?

Yes

Is the case presented with sufficient detail to be useful for other practitioners?

Partly

Is sufficient discussion included of the importance of the findings and their relevance to future understanding of disease processes, diagnosis or treatment?

Partly

Is the background of the case’s history and progression described in sufficient detail?

Yes

Reviewer Expertise:

Expertise in basic biology of Wilson Disease and working knowledge of diagnostic procedures.

I confirm that I have read this submission and believe that I have an appropriate level of expertise to confirm that it is of an acceptable scientific standard.

# Response

**Published:** 2022-01-14

**Authors:** Sudeep Adhikari

Thank you for the review, and the constructive feedback.

# Peer review: Reviewer response for version 1

**DOI:** 10.5256/f1000research.58069.r89924

**Published:** 2021-08-09

**Authors:** Raffaele Iorio

**Affiliations:** 1 Department of Translational Medical Science, Section of Pediatrics, University of Naples Federico II, Naples, Italy

Since screening for rare diseases is not always possible in low-income countries, with this case report the authors emphasize the usefulness of the ‘Wilson’s pattern’ LFT as a guide for suspecting Wilson’s disease in patients with liver disease. Certainly the case report is of value if we contextualize it and if we take into account the error of easily attributing patients' liver disease to alcohol without conducting an appropriate differential diagnosis. However, the message of the studies itself is very limited and not original. As the authors themselves point out, sensitivity and the specificity of this model has yet to be validated in patients with subacute liver disease so the authors' observation, although simulating, cannot be considered as a sure proof of the diagnostic reliability of the test. Certainly the authors should be congratulated for the diagnostic suspicion.

 Regarding the patient's definition of negative Coombs hemolytic anemia, which gave her a score of 1 according to the Leipzig score, I believe that for the definition of hemolysis it was necessary to have the result of the reticulocytes. In other words, to be defined haemolytic anemia required other parameters (reticulocytes, indirect bilirubin, haptoglobin, etc).

 Given the presence of cirrhosis and ascites, a comment should be done  on the choice of therapy with zinc instead of chelating.

Are enough details provided of any physical examination and diagnostic tests, treatment given and outcomes?

Partly

Is the case presented with sufficient detail to be useful for other practitioners?

Yes

Is sufficient discussion included of the importance of the findings and their relevance to future understanding of disease processes, diagnosis or treatment?

Partly

Is the background of the case’s history and progression described in sufficient detail?

Partly

Reviewer Expertise:

Liver disease, Wilson' disease, pediatric liver disease

I confirm that I have read this submission and believe that I have an appropriate level of expertise to confirm that it is of an acceptable scientific standard, however I have significant reservations, as outlined above.

# Response

**Published:** 2022-01-14

**Authors:** Sudeep Adhikari

Thank you for the constructive feedback.

 Changes have been made in the revised version regarding the issues raised.

 Regarding Coombs negative hemolytic anemia, details regarding indirect bilirubin and reticulocyte count have been added, however haptoglobin level was not tested.

 Regarding choice of therapy with zinc acetate instead of d-penicillamine, the fact that the d-penicillamine was not available for use has been acknowledged in the revised manuscript.
